# Optimising multistage dairy cattle breeding schemes including genomic selection using decorrelated or optimum selection indices

**DOI:** 10.1186/1297-9686-44-1

**Published:** 2012-01-17

**Authors:** Vinzent Börner, Norbert Reinsch

**Affiliations:** 1 Leibniz Institute for Farm Animal Biology, Wilhelm-Stahl-Allee 2, 18196 Dummerstorf, Germany

## Abstract

**Background:**

The prediction of the outcomes from multistage breeding schemes is especially important for the introduction of genomic selection in dairy cattle. Decorrelated selection indices can be used for the optimisation of such breeding schemes. However, they decrease the accuracy of estimated breeding values and, therefore, the genetic gain to an unforeseeable extent and have not been applied to breeding schemes with different generation intervals and selection intensities in each selection path.

**Methods:**

A grid search was applied in order to identify optimum breeding plans to maximise the genetic gain per year in a multistage, multipath dairy cattle breeding program. In this program, different values of the accuracy of estimated genomic breeding values and of their costs per individual were applied, whereby the total breeding costs were restricted. Both decorrelated indices and optimum selection indices were used together with fast multidimensional integration algorithms to produce results.

**Results:**

In comparison to optimum indices, the genetic gain with decorrelated indices was up to 40% less and the proportion of individuals undergoing genomic selection was different. Additionally, the interaction between selection paths was counter-intuitive and difficult to interpret. Independent of using decorrelated or optimum selection indices, genomic selection replaced traditional progeny testing when maximising the genetic gain per year, as long as the accuracy of estimated genomic breeding values was ≥ 0.45. Overall breeding costs were mainly generated in the path "dam-sire". Selecting males was still the main source of genetic gain per year.

**Conclusion:**

Decorrelated selection indices should not be used because of misleading results and the availability of accurate and fast algorithms for exact multidimensional integration. Genomic selection is the method of choice when maximising the genetic gain per year but genotyping females may not allow for a reduction in overall breeding costs. Furthermore, the economic justification of genotyping females remains questionable.

## Introduction

Genomic selection (GS) offers breeders the opportunity to reduce costs, decrease the generation interval [1] and possibly avoid inbreeding [2]. GS is based on the prediction of breeding values from individual genotypes (estimated genomic breeding values, GEBV). These genotypes consist of a large number of DNA markers in the form of single nucleotide polymorphisms (SNP), which are in linkage disequilibrium with quantitative trait loci coding for economically important traits.

In dairy cattle, accuracies (r) of GEBV(r_QEBV_) for milk production traits can be as high as 0.75 [3], but are below those from progeny testing. However, the tremendously decreased generation interval (L) may lead to a much higher genetic gain per year (Δ G_a_) [1,4]. Schaeffer [1] summarised the potential effects of GS on dairy cattle breeding schemes assuming an rGEBV of 0.75 and a cost for GS per genotyped individual (CQEBV) of 500 Canadian Dollars in a one-stage selection approach. As rGEBV and CQEBV may change, research work concerning optimum breeding schemes for different combinations of these parameters and possible multistage selection approaches are of interest.

The availability of a variety of SNP chips at different prices and yielding breeding values of different accuracies makes it possible to use specific SNP chips in each selection path or more than one chip in a multistage preselection approach. Additionally, the advantage of GS might also be combined with traditional progeny performance testing (PPT) schemes as currently applied in certain dairy cattle breeding programs [5]. Thus, apart from the already answered questions concerning the applicability of GS, the answer to the question of how a breeding scheme should be structured has become more complex. This complexity results from the possibility of combining different information sources according to their costs and correlations with the aggregate genotype, allowing for a variety of one-, two- or multistage breeding schemes in each selection path to choose from.

It is a question of economic optimisation to select the breeding scheme which maximises a defined utility function. In multistage breeding schemes, the information about the selection candidates collected at all previous stages is combined with the information collected at the current stage. Therefore, the estimated breeding values (EBV) of successive stages are correlated and the EBV of selected individuals after the first selection stage are non-normally distributed. One of the major challenges of research on optimising multistage breeding schemes is the necessity of using computationally sophisticated multiple integration techniques to derive the selection intensities. When Ducrocq and Colleau [6] applied such methods on multistage dairy cattle breeding schemes they were faced with the problem that the convergence of such algorithms can be difficult to achieve if the EBV of successive stages are highly correlated. Furthermore, the computational time was seen as unacceptable if the number of selection stages became too high [7].

A possible solution to circumvent these problems is the decorrelation of the EBV of successive stages, as proposed by Xu and Muir [8,9]. Then, a normal distribution can be assumed for EBV at each stage, making it easy to calculate selection intensities. Xu and Muir [9] estimated the loss in the predicted genetic gain due to the decorrelation in a two-stage breeding scheme to be up to 10% compared to the exact calculation solving the integral. This loss was justified by the possibility of implementing an unlimited number of stages, which was otherwise not applicable.

Decorrelated indices have been used for model calculations of breeding schemes for poultry [9,10], beef cattle [11] and marker-assisted selection [12]. In all these applications accuracies of breeding values, selection intensities and generation intervals across selection paths were assumed identical. Thus, the applicability of decorrelated indices for complex breeding schemes has not been investigated in detail regarding a) different selection paths, b) their interaction due to the effects of the selection strategy in one path on the accuracy of EBV of the other paths, c) different selection intensities and EBV accuracies in each path, d) the interaction between the generation interval and the number and intensities of successive selection stages and, finally, e) the opportunity to split financial investments between selection paths.

Numerical integration techniques developed more recently by Genz [13], in conjunction with the maximisation algorithm of Brent [14], allow for a fast and stable calculation of the exact selection intensities, even when using many stages. The aim of our work was to compare the results of breeding scheme optimisations when the approach of Xu and Muir [9] (decorrelated indices) or numerical integration (optimum indices) was used to derive selection intensities, breeding value accuracies, and Δ G_a_. Both methods were applied to a complex dairy cattle breeding scheme as mentioned above. The possibility of using GS as an optional selection stage in a way that it can be used in addition to or instead of PPT was allowed for. Optimisation was over a semi-continuous parameter range of r_GEBV_and C_GEBV_and financial resources were restricted. Therefore, the results provide insights into the sensitivity of dairy cattle breeding plans to variation in these parameters.

## Methods

### Construction of selection indices and the implementation of GEBV

Deterministic methods were used to optimise breeding plans. Accuracies of EBV (r_EBV_) were calculated based on the selection index theory and coefficients for regressing the aggregated genotype of the selection candidate on phenotypic measurements of informants were derived using two different methods.

The GEBV was included in the selection index as a trait with a heritability of one and a genetic correlation determined by its accuracy [15,16].

For the optimum selection indices (OSI), standard selection index methodology was used at each selection stage *i *= 1,.., *m*_*j *_of each selection path *j *= 1,.., *n*.

For decorrelated selection indices (DSI) the regression coefficients ***b***_*ij *_were constructed according to Xu and Muir [9]:

(1) $$\begin{array}{ll} b_{ij} & =P_{ij}^{-1}G_{ij}w\;\\ 0 & ={b\prime }_{ij}P_{i\left(i-1\right)j}B_{\left(i-1\right)j}\left(i>1\right)\;\\ 1 & ={b\prime }_{ij}P_{ij}b_{ij}\;\\ & \; \end{array}$$

where ***w ***is the vector of economic weights of the traits in the aggregated genotype, ***b***_*ij *_is the vector of regression coefficients on all available information sources, ***G***_*ij *_is the genetic-phenotypic covariance matrix, ***P***_*ij *_is the phenotypic covariance matrix of all information sources up to stage *i*, ***P***_*i*(*i*-1)*j *_is the phenotypic covariance matrix of all information sources up to stage *i *-- 1 and ***B***_(*i-*1)*j *_is a matrix of regression coefficients for all stages previous to *i*. Note that second and third equations are used as constraints when solving the first equation, and are incorporated into it via Lagrange multipliers. The first constraint guarantees that the covariance between EBV of stage *i *and EBV of all other stages is equal to zero and the second that a solution exists.

**Figure 1:**
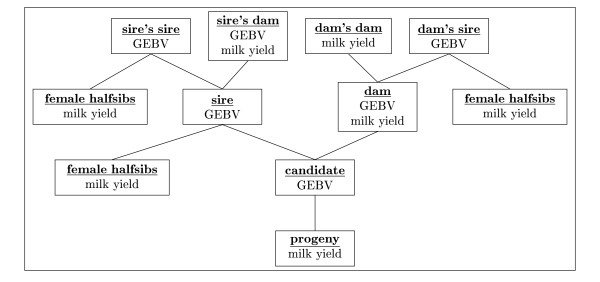
**Standard pedigree used to derive breeding values in all selection paths**.

The pedigree and associated phenotypic and genomic information for candidates and relatives available to construct case specific selection indices are given in Figure 1.

### Genetic gain

The genetic gain of each path (Δ G_j_) was calculated differently depending on whether DSI or OSI was used. When using OSI, the following formula was applied:

(2) $$\Delta G_{j}=z_{ij}\sigma_{a}$$

where *z*_*ij *_is the expectation of the aggregated genotype (selection intensity) of the selected individuals after selecting at stage *i *(the last stage in path *j*) and *σ*_*a *_is the standard deviation of the aggregated genotype. Selection intensities were derived using the moment generating function of a truncated multivariate normal distribution including all used selection stages and the aggregated genotype [17], where the truncation points were calculated following the approach of Mi and Utz [18], merging the integration algorithm of Genz [13] and the maximisation techniques of Brent [14].

Using the decorrelated index approach of Xu and Muir [9], Δ G_j _was calculated avoiding multiple integration according to

(3) $$\Delta G_{j}=\sum_{i=1}^{m_{j}}z_{ij}\sigma_{ij}$$

where *z*_*ij *_is the selection intensity and *σ*_*ij *_is the standard deviation of the decorrelated EBV. Due to the zero covariance between EBV of successive stages, *z*_*ij *_could be calculated by one-dimensional integration.

Δ G_a _for the whole breeding scheme was calculated according to Rendel and Robertson [19]:

(4) $$\Delta G_{a}=\frac{\sum_{j=1}^{n}\Delta G_{j}}{\sum_{j=1}^{n}L_{j}},$$

where *L*_*j *_is the generation interval in path *j.*

### Breeding program

A cooperative Holstein dairy cattle breeding program with a cow population of 100 000 was used. Bull dams were selected as heifers from all available heifers and were assumed to be used as bull dams only once. Male selection candidates were produced by contract mating to bull dams. For the purpose of comparing methods, only one milk trait with a heritability of 0.25 was set in the breeding goal. Selection took place on EBV combining performance data and GEBV.

The biological, technical and economical parameters of the program are given in Table 1. All parameters of the breeding program that are expressible in terms of probabilities and not given in Table 1, such as success of first insemination, survival rate of calves, etc., were assumed to be zero or one.

**Table 1:** Biological, economical and technical parameters of the breeding program

parameter	unit	value
*h*^2 ^milk trait		0.25
phenotypic standard deviation	kg	700
accuracy GEBV^1^		0.3 - 0.9
age at first calving	month	26
time between calving	month	12
length of lactation	month	10
maturity of test bulls	month	14
number of daughters per sire	head	99^3^
insemination with test bulls	%	20
average age of bull calves at purchase	month	6
price of bull calves	**€**	4 000
keeping costs of bull calves until maturity	**€**/day	5
keeping costs of test bulls	**€**/day	4.5
cost for genomic selection^2^	**€**	25 - 400
population of cows	head	100 000
demand for cow sires	head/year	10
demand for bull sires	head/year	5
initial male selection candidates	head/year	500
demand for bull dams (contract matings)	head/year	1000
compensation payments for test bull matings	**€**/test bull	3 000
compensation payments to breeders for keeping genotyped selection candidates	**€**/candidate	150
maximum breeding program costs	**€**/year	719 050
L_ss_^4 ^(PED^5^, GS^6^, PPT^7^)	months	23, 23, 71
L_S_D^8 ^(PED, GS, PPT)	months	23, 23, 61
L_DS_^9 ^(PED, GS)	months	26, 26

1: estimated genomic breeding values, 2: per genotyped individual, includes DNA isolation, genotyping and calculation of breeding values, 3: minimum number, recalculated according to the number of bulls entering the progeny testing stage, 4: generation interval in the path "sire-sire" as a function of the final selection stage, 5: selection stage selecting on ancestor and sib performance and GEBV, 6: selection stage selection on candidate's GEBV, 7: selection stage selecting on candidate's progeny performance, 8: generation interval in the path "sire-dam" as a function of the final selection stage, 9: generation interval in the path "dam-sire" as a function of the final selection stage

The selection paths were "sire to sire"(SS), "sire to dam"(SD), "dam to sire"(DS) and "dam to dam" (DD). Since in practise, almost no selection takes place within the path DD, a selection intensity of zero was assumed for this path. Each selection path was structured in stages. The selection stages available in paths SS and SD were a) selection on performance data and GEBV of ancestors and half-sibs (PED stage), b) selection on the candidate's GEBV (GS stage) and c) selection on progeny performance data (PPT stage). The selection stages available in path DS were a) selection on performance data and GEBV of ancestors and half-sibs (PED stage), and b) selection on the candidate's GEBV (GS stage).

To genotype and select male calves, a tissue sample was taken by a veterinarian on farm at birth and had to be paid for by the farmer. DNA-isolation and SNP-genotyping was carried out by a central laboratory, and was added to the expenses of the breeding organisation. Selection candidates were kept on farm until the age of six months. Farmers keeping male candidates selected at the PED stage and slaughtered after GS received a compensation payment from the breeding organisation. When genotyping female calves as potential bull dams, costs were similar, but since the bull dams were not bought by the breeding organisation, no compensation was paid.

### Breeding cost

Costs not related to selection strategies (e.g. performance recording of females, calculating EBV, marketing, semen processing) were not considered. To allow for changes in costs for labour and infrastructure due to different breeding schemes, all expenses were derived from some invariant basic cost given in Table 1 via the cost function of NamKoong [20]:

(5) $$C_{bj}=C_{n}n_{j}\prod_{k=1}^{i-1}p_{jk},$$

where *C*_*bj *_is the total cost of expense factor *b *in path *j, C*_*b *_is the cost of expense factor *b *per individual, *n*_*j *_is the number of initial selection candidates, *p*_*jk *_is the proportion of the available individuals that is selected at stage *k *and *i *is the stage within path *j *at which the selection stops concerning *C*_*b*_*. C*_*b *_can be costs for genotyping, purchasing, keeping the animals until maturity, keeping them from maturity until their breeding value is estimated from PPT, compensations for test bull insemination, or compensations for keeping the animals during genotyping stages.

Note that in the case of compensation for keeping the animals during genotyping, the cost formula changes to

(6) $$C_{bj}=C_{b}n_{j}\prod_{k=1}^{i-1}p_{jk}\left(1-p_{ji}\right).$$

The breeding costs of path *j *were the sum over all expense factors in the path, and the total cost for a certain breeding program was the sum over all paths.

The maximum breeding cost of 719 050 **€ **was imposed as a constraint during maximisation and was derived assuming progeny performance testing of 50 young bulls per year:

(7) $$\begin{array}{l} (4000€+213.5\text{d}\times 5€/\text{d}+\\ 1403\text{d}\times 4.5€/\text{d}+3000€)\times 50. \end{array}$$

This cost included the purchase of 50 male calves from contract matings, their maintenance until maturity and from maturity until breeding value estimation from PPT, and compensation payments for test bull insemination.

### Parameter variation

Various values for C_QEBV_and r_QEBV_were used during the calculation process, rGEBV varied between 0.3 and 0.9 in steps of 0.025 and CQEBV between 20 **€ **and 400 **€ **in steps of 10 **€**, resulting in 975 combinations of r_GEBV_and C_QEBV_.

### Maximisation

Breeding schemes maximising Δ G_a _for each combination of C_QEBV_and r_GEBV_were obtained using a grid search in which the proportion of selected individuals varied at each selection stage in every path. Within the maximisation process, trait measurements available for ancestors, and, therefore the accuracy of the candidates' EBV were adjusted according to the selection strategy in the path from which the ancestor had been derived. Furthermore, to select bull sires from cow sires, the possibility of additional selection stages was allowed for, which requires additional information (e.g. PPT stage) instead of just increased selection intensity for the same group of males.

For each path, the initial number of selection candidates and the final number of selected individuals for reproduction were fixed. The product of the selected proportions at each stage *i *within path *j *(*p*_ij_) had to fulfil the equation:

(8) $$\frac{s_{j}}{n_{j}}=\overset{m_{j}}{\underset{i=1}{\prod }}p_{ij},$$

where *s*_*j *_is the number of selected individuals actually used for reproduction and *m*_*j *_is the number of selection stages in path *j.*

The proportion *p*_ij _varied between 0.01 and 1 in steps of 0.025. Stages with *p*_*ij *_= 1 were treated as not used. For the stages used (*p*_*ij *_< 1), the constraint of equation 8 was fulfilled by calculating *p*_*ij *_of the last used stage as a dependent variable. Only if this value was ≤ 1, was the stage combination considered as valid. The valid stage combinations of all paths were completely cross-classified to obtain all possible breeding schemes. Breeding costs were derived for each of these schemes but Δ G_a _was only calculated if the cost constraint was fulfilled. The breeding scheme with the highest Δ G_a _was seen as the optimum for the given combination of C_QEBV_and r_QEBV_.

For each combination of r_QEBV_and C_QEBV_, a grid of about 60 000 breeding schemes was searched for optimisation. The total amount of evaluated breeding plans was 58 million for each calculation method, OSI and DSL

All calculations were carried out with a FORTRAN 90 program written by the first author. The calculation of the selection intensity of an optimum index in a multistage breeding program used the FORTRAN routines of Genz [21] and Brent [22].

## Results

### Comparison of methods to calculate genetic gain

**Figure 2:**
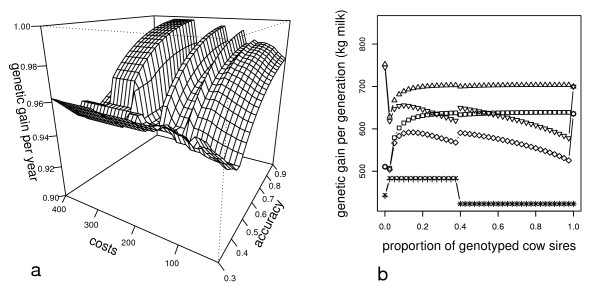
**Comparison of results when decorrelated (DSI) or optimum selection indices (OSI) were applied**. Figure 2(a) shows the genetic gain per year from applying DSI as a proportion of the one from applying OSI. Figure 2(b) shows the genetic gain per generation of different selection paths of breeding schemes that maximise the genetic gain per year given an accuracy and cost of estimated genomic breeding values of 0.75 and of 150 **€**, respectively, when the proportion of genotyped males is varied along the x-axis and all other parameters were chosen such that the genetic gain per year was maximised; paths "sire-sire" (Δ) "sire-dam" (□) and "dam-sire" (+) calculated with optimum indices, and of the paths "sire-sire" (∇), "sire-dam" (◊) and "dam-sire" (x) calculated with decorrelated indices

Parameters and results of the breeding schemes that maximise Δ G_a _and fulfill the cost constraint were compared between OSI and DSL Table 2 summarises the frequency of certain selection strategies of optimum breeding schemes in paths SS, SD and DS as a function of TGEBV and the application of either OSI or DSL For selection in path SD with rQEBV ranging from 0.3 to 0.4, both methods found that combined selection at PED and GS stage or a three-stage selection approach maximised Δ G_a_. The same holds for path SS for an rQEBV range between 0.3 and 0.45. Beside these similarities, applying DSI also led to strategies for male selection that did not include GS, which was never the case when using OSI. Furthermore, a three-stage selection approach in paths SS and SD was found to maximise Δ G_a _much more frequently when using OSI. The differences between the proposed selection strategies in path SD are even more obvious within r_GEBV_ranging between 0.45 and 0.9, including 741 possible parameter combinations. When using OSI, in all these cases the breeding schemes that maximised Δ G_a _used two-stage selection of sires, whereas when using DSI in 680 cases one-stage selection of sires was found to be optimum. Similar results were obtained for path DS, where two-stage selection was the most frequent strategy that maximised Δ G_a _when using OSI compared to DSI. Except for one-stage selection procedures, DSI always suggested breeding schemes yielding less Δ G_a _than OSI, with a maximum reduction of 5.5% and a mean of 2% (see Figure 2(a)). If all optimization results obtained by applying DSI were recalculated using OSI, which means using the selection intensities obtained from DSI and using these with OSI, the loss in the predicted Δ G_a _due to DSI was up to 5.5%, and up to 7% for the predicted Δ G_SS_(results not shown). For the reverse recalculation, i.e. using selection intensities of optimum breeding schemes obtained from OSI in DSI, the loss in the predicted Δ G_a _increased up to 29%, and the loss in the predicted Δ G_SS_up to 40% (results not shown).

**Table 2:** Number of different selection strategies in each selection path

path	${\text{r}}_{\text{GEB}{\text{V}}^{1}}$	n^2^	$n_{Pd^{3}}$		$n_{Pd+PT^{4}}$		$n_{Pd+GS^{5}}$		$n_{Pd+GS+PT^{6}}$		$n_{GS^{7}}$	
			
			DSI^8^	OSI^9^	DSI	OSI	DSI	OSI	DSI	OSI	DSI	DSI
SD^10^	0.3-0.4	195	0	0	120	0	23	22	52	173	0	0
SS^11^	0.3-0.45	273	0	0	120	0	30	25	123	248	0	0
SD	0.45-0.9	741	0	0	0	0	61	741	0	0	680	0
DS^12^	0.425-0.5	156	0	0	0	0	156	156	0	0	0	0
DS	0.3-0.9	975	221	121	0	0	754	854	0	0	0	0

Number of different selection strategies in each selection path of breeding schemes which maximised Δ G_a _and the accuracy of estimated genomic breeding values. 1: parameter space for the accuracy of estimated genomic breeding values, 2: number of possible breeding schemes within the given parameter space, 3: number of schemes with selection only on pedigree data, 4: number of schemes with selection on pedigree data and progeny performance, 5: number of schemes with selection on pedigree data and estimated genomic breeding values, 6: number of schemes with selection on pedigree data, estimated genomic breeding values and progeny performance, 7: number of schemes with selection only on estimated genomic breeding values, 8: decorrelated indices are used, 9: optimum indices are used, 10: selection path "sire-dam", 11: selection path "sire-sire", 12: selection path "dam-sire", the parameter space for the cost of genomic selection per genotyped individual is 20-400 **€**

A key to understand the different results when using DSI or OSI is given in Figure 2(b) and Table 3. Decorrelating EBV of subsequent selection stages is equal to decomposing e.g. the variance at stage 2, $\sigma_{2}^{2}$, into $\sigma_{1}^{2}+2\sigma_{1,2}+\sigma_{e}^{2}$ (the $\sigma_{1}^{2}$ is the variance at the previous stage and *σ*_1,2 _is the respective covariance) and reducing the variance of the information source at stage 2 to $\sigma_{e}^{2}$. Thus, an increase in the covariance between stages leads to a further reduction of the variance of EBV in the last selection stage. This is exemplified in the last two rows of Table 3. Including a GS stage in path DS increased the accuracy of the PED stage EBV and the covariance between PED stage and GS stage in path SD, but led to a reduction of the accuracy of the GS stage EBV in this path to a value below that of the PED stage EBV. The effect of this interaction on Δ G is given in Figure 2(b), which reflects Δ G_SD_, Δ G_SS_and Δ G_DS_of breeding schemes that maximized Δ G_a _as a function of DSI, OSI and the proportion of genotyped initial male selection candidates (PGsd) given an rQEBV of 0.75 and Cqebv of 150 **€**. All other parameters, e.g. the number of genotyped females were chosen such that Δ G_a _was maximised. If PGsd was set to zero, a breeding scheme selecting males at PED and PPT stages and females only at the PED stage was found to maximise Δ G_a _(GS of females was not possible due to cost limitations). In this case, Δ G_DS_of both methods were equal because of the one-stage selection approach. The difference in Δ G_SD_and Δ G_SS_was small because of the high selection intensity at the PED stage and a DSI induced reduction of the accuracy of the PPT stage EBV from 0.993 to 0.814. If genotyping was allowed, as indicated by PG_SD_> zero, breeding schemes that maximised Δ G_a _excluded the PPT stage in paths SD and SS in favour of inclusion of a GS stage in all paths. GS of females had to be abandoned due to cost constraints if PG_SD_exceeded 0.375. The DSI induced reduction of the accuracy of GS stage EBV below that of PED stage EBV led to a strong peak of Δ G_SS_and Δ G_SD_at an already low PG_SD_value. Furthermore, abandoning pre-selection at the PED stage (PG_SD_equal to one) led to a sharp increase in Δ G_ss _and Δ G_SD_because one-stage selection made decorrelation unnecessary. The counter-intuitive interaction between paths due to DSI became even more obvious if GS of females was abandoned, which slightly decreased Δ G_SD_and Δ G_SS_when OSI was applied but increased these genetic gains when DSI was applied. This resulted from an inversion of the process well described in the last two rows of Table 3. Thus, the reason behind the given interaction is the characteristic of DSI to exploit only the residual variance. This can reduce the accuracy of EBV of a certain stage below those of the preceding stages, which leads to a loss of Δ G_a _if two- or multi-stage selection is applied. Additionally, more accurate EBV in one path can have negative side effects on EBV accuracies and Δ G in other paths. Such results are impossible when using OSI.

**Table 3:** The accuracy of estimated breeding values of successive selection stages, ${\text{r}}_{\text{GEB}{\text{V}}^{1}}=0.75$

Selection path	Selection stage		Accuracy of estimated breeding values by stage			
			
			Optimum indices		decorrelated indices	
			
	PED^2^	GS^3^	PED	PED + GS	PED	GS
SD^4^	yes	no	0.177	-	0.177	-
DS^5^	yes	no	0.347	-	0.347	-

SD	yes	no	0.427	-	0.427	-
DS	yes	yes	0.379	0.766	0.379	0.606

SD	yes	yes	0.441	0.754	0.441	0.612
DS	yes	no	0.499	-	0.499	-

SD	yes	yes	0.537	0.754	0.537	0.530
DS	yes	yes	0.50	0.773	0.50	0.589

The accuracy of estimated breeding values of successive selection stages as a function of the implementation of genomic selection and the used selection indices. 1: accuracy estimated genomic breeding value, 2: selection on performance and GEBV of ancestors and sibs, 3: selection on candidates own GEBV, 4: selection path "sire to dam", 5: selection path "dam to sire"

### Genetic gain of optimum indices

**Figure 3:**
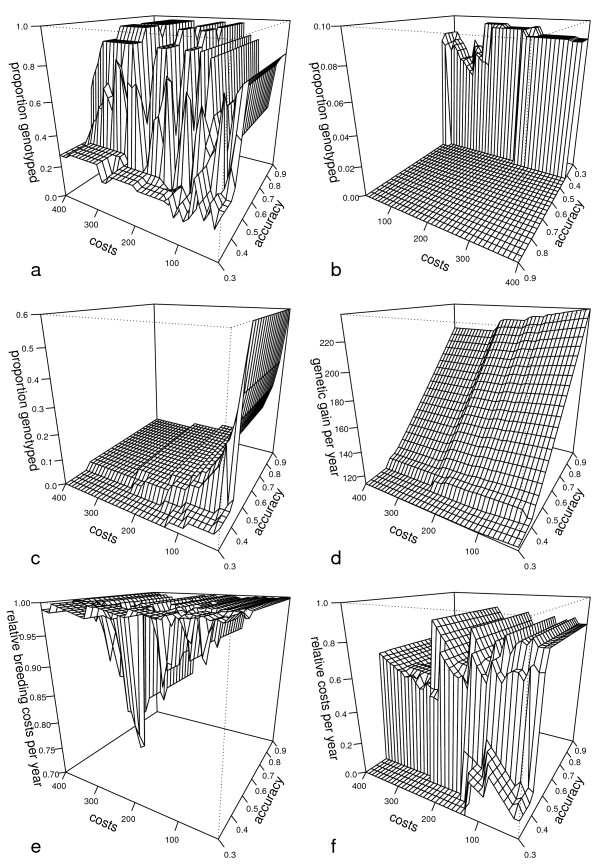
**Characteristics of breeding schemes that maximise the genetic gain per year when optimum selection indices were used**. 3(a) Proportion of genotyped initial selection candidates in the sire-dam path 3(b) Proportion of progeny tested initial selection candidates in the sire-dam path 3(c) Proportion of genotyped initial selection candidates in the dam-sire path 3(d) Genetic gain per year 3(e) Total breeding cost as proportion of the maximum cost 3(f) Breeding cost of the dam-sire path as proportion of total breeding cost

Results when using OSI are given in Figure 3. Δ G_a _increased with increasing r_GEBV_but was almost independent of C_GEBV_. The highest Δ G_a _of 236.94 kg or 0.67 genetic standard deviations was achieved at a parameter combination of r_QEBV_= 0.9 and C_GEBV_= 20 **€**, whereas the lowest Δ G_a _of 113.09 or 0.32 genetic standard deviations was achieved at a parameter combination of r_QEBV_= 0.3 and C_GEBV_between 380 and 400 **€**.

The relative contribution of the different selection paths to the total genetic gain of breeding schemes was between 0.46 and 0.34 for path SS, between 0.36 and 0.29 for path SD, and between 0.35 and 0.19 for path DS. Only in 21 of 975 parameter combinations did the relative contribution of path DS exceed that of path SS, but by no more than 0.01. The relative contribution of path SD was exceeded by that of path DS in 202 of 975 cases; wherein the maximum excess was 0.04. Thus, in the vast majority of parameter combinations, the main contribution to genetic gain came from path SS (results not shown).

The total cost of breeding schemes that maximised Δ G_a _ranged from 544 685 to 718 973 **€ **but only in 218 of the 975 parameter combinations was it below 700 000 **€**. Breeding costs incurred by genotyping females ranged from zero (no genotyping) to > 90% of the total breeding costs but was greater than 50% in 802 of the 975 parameter combinations. As given in Figure 3(f), a prerequisite for such a high proportion of total cost from genotyping females was an r_QEBV_> 0.4. Below this value, the marginal benefit of a reduced selection intensity at the PED stage and an increased selection intensity at the GS stage in path DS was not found to maximise Δ G_a_.

The highest proportion of genotyped bull dams i.e. 0.6, which equals 30 000 heifers, was found when genotyping costs were the lowest and r_GEBV_was ≥ 0.45. This proportion decreased with increasing C_GEBV_, but GS was applied to select females in almost all parameter combinations except when C_GEBV_exceeded 120 **€ **in conjunction with an r_GEBV_range between 0.3 and 0.4. As PG_SD_was always below one, selection in paths SS and SD included a PED and a GS stage if r_GEBV_ranged between 0.45 and 0.9 and between 0.4 and 0.9 for bull sires and cow sires, respectively. Below an r_GEBV_of 0.4(SD) and 0.45(SS) three-stage selection strategies with a PPT stage were also found to maximise Δ G_a_. Furthermore, for r_GEBV_between 0.425 and 0.45, bull sires were selected from cow sires on the basis of an additional PPT stage. Nevertheless, a pure two-stage selection strategy with only a PED and a PPT stage was not found to maximise Δ G_a _across all 975 parameter combinations.

## Discussion

### Comparison of the results for the decorrelated and optimum index

The results of this study quantify the respective loss in predicted Δ G_a _from using DSI instead of OSI to be up to 5.5% and in Δ G per generation to be up to 6%. This is within the range given by Xu and Muir [9]. Nevertheless, DSI changes the functional dependencies of selection intensity and Δ G as well as Δ G_a_. Although the modelled breeding scheme was less complex, tracing the interaction among selection stages and paths was quite difficult. This might become impossible if DSI is applied to more complex breeding schemes with numerous selection stages in a variety of paths with different selection intensities and generation intervals. Thus, DSI has its mathematical intrinsic logic, but for breeders its results are difficult to interpret, counter-intuitive or suboptimal.

The genetic variance is known to be reduced by the "Buhner" effect, selection-induced gametic disequilibrium [23], leading to an overestimation of asymptotic rates of genetic response [24-26] if this effect is not accounted for as was the case in our study. The ranking and relative differences between alternative breeding programs have, however, been found to be little affected by ignoring this effect [24,25]. Comparisons between OSI and DSI-types of selection indices are not affected, because ignoring the Bulmer effect is equivalent to a comparison in terms of one-generation responses.

One of the advantages of decorrelated indices mentioned by Xu and Muir [9] is the ability to use maximisation methods that use first and second derivatives of the goal function. However, this option is limited to breeding schemes with equal generation intervals because otherwise the goal function might change in a non-continuous manner with the number of selection stages. Furthermore, the cost function of NamKoong [20] is not continuous if the selection intensities of certain selection stages converge to zero in the maximisation process. Thus, grid searches or heuristic approaches are still the methods of choice when goal functions are difficult or impossible to differentiate or non-continuous. As limitations due to central processing unit time have almost been overcome because of developments in efficient hardware and fast algorithms [13], the exact calculation of optimum indices in combination with the above mentioned methods is the better alternative.

### Results using the optimum index

In this study, we found for a given TGEBV of 0.75, a Δ G_a _between 0.53 and 0.57 genetic standard deviation, which is higher than found in other published results [1,27]. This may result from differences in the underlying selection intensities in paths SS and SD, which were higher in our calculations. As already mentioned, the effect of selection on genetic variance in later generations ("Bulmer" effect) was not accounted for. However, the results are still comparable to other studies that did not consider this effect either [1,28]. Furthermore, since ranking the breeding schemes might not be affected, the main conclusion remains the same. Ignoring reduction in variances in later stages also affects optimal breeding schemes in terms of the proportion selected in each stage and whether or not a given stage is utilised. So including this effect is important, which our methods do.

The strong fluctuation of PG_SD_among programs that maximise genetic gain (see Figure 3(a)) can be explained by the non-linearity of Δ G as a function of selection intensity (see the OSI curves in Figure 2(b)). In optimum breeding schemes, PG_SD_was mostly at its upper limit, where the marginal benefit or loss in Δ G_a _from a small increase or decrease of selection intensity was very small. On the contrary, due to the imposed cost constraint, the proportion of genotyped females was small, leading to a high sensitivity of Δ G_DS_to any change in selection intensity. In the case of increasing C_GEBV_three scenarios were possible. A: Cost was increased as long as the number of genotyped females could be maintained by reducing PG_SD_with negligible effect on Δ G_a_. B: Cost was increased until a reduction of PG_SD_let to a loss in Δ G_SD_being higher than a loss in Δ G_DS_due to reduced genotyping of females. C: Cost was increased till even not genotyping males could not save enough money to maintain the number of genotyped females. In the latter two scenarios the number of genotyped females had to be decreased and the available funds could be reinvested to increase PG_SD_up to the maximum achievable Δ G_SD_.

Schaeffer [1] found that path DS became the main source of genetic gain but he assumed that for each possible bull dam a highly accurate GEBV was estimated. However, when calculating the breeding costs for such a GS scheme, genotyping costs for only 2000 bull dams were regarded. In our breeding programs genotyping large proportions of the population of potential bull dams was not possible due to cost limitations. Thus, paths SS and SD were generally the main sources of Δ G_a_. Furthermore, genotyping bull dams was the major source of breeding costs, leading to values higher than 90% of the maximum possible breeding costs in the majority of parameter combinations. This exemplifies a trade-off between decreased overall breeding costs and the importance of path DS for Δ G.

Since implementing GS allows to gather information on selection candidates relatively cheaply compared to PPT, the selection intensity can be increased because of a higher number of selection candidates. When doing so, breeding organisations should take into consideration that the additional Δ G_a _from an extended selection basis is approaching zero. Additionally, optimising breeding schemes should also include the cost of the invested capital and Δ G_a _per **€**. Such parameters may not allow for the inclusion of GS in path SD, as given in our results and for the application of GS in general in path DS, and may also question the utility of genotyping even whole sub-populations, as proposed by König and Swalve [28].

Some breeding organisations rely on using GS as a preselection stage followed by PPT [5]. The continuation of progeny testing in combination with GS was found to be economic only at an TGEBV ≤0.4 in path SD and ≤0.45 in path SS. Since an r_QEBV_of 0.7 can be achieved in practical breeding programs [29], there may be no alternative to replacing conventional progeny testing by GS in order to maximise the genetic gain per year.

## Conclusions

Applying decorrelated indices to multistage dairy cattle breeding schemes including genomic selection in an optimisation approach taking into consideration the strong interaction between selection paths led to results that were not only difficult to interpret but also counter-intuitive or suboptimal. This may result in improper advice to breeding organisations. Since fast and stable calculation of selection intensities in multistage breeding programs is possible even for highly correlated EBV of successive stages and small proportions of selected individuals, the optimum selection index is the method of choice for the deterministic optimisation of breeding schemes using the selection index methodology.

Genomic selection might meet its promises concerning the increase in genetic gain per year, although the effects on breeding costs are still unclear. However, the relatively low financial efforts to obtain estimated genomic breeding values compared to progeny performance testing make it possible to optimise breeding schemes in a holistic across-path approach, which also includes the risk of losing money due to opportunity costs.

## Competing interests

The authors declare that they have no competing interests.

## Authors' contributions

VB worked out the conception and design of the simulation study, wrote computer program and analysed the results and wrote the article. NR worked out the conception and design of the simulation study and revised the afcle **All authors have read and approved the final manuscript**.
